# Tracking Seed Fates of Tropical Tree Species: Evidence for Seed Caching in a Tropical Forest in North-East India

**DOI:** 10.1371/journal.pone.0134658

**Published:** 2015-08-06

**Authors:** Swati Sidhu, Aparajita Datta

**Affiliations:** Eastern Himalaya Programme, Nature Conservation Foundation, Mysore, Karnataka, India; Estación Biológica de Doñana, CSIC, SPAIN

## Abstract

Rodents affect the post-dispersal fate of seeds by acting either as on-site seed predators or as secondary dispersers when they scatter-hoard seeds. The tropical forests of north-east India harbour a high diversity of little-studied terrestrial murid and hystricid rodents. We examined the role played by these rodents in determining the seed fates of tropical evergreen tree species in a forest site in north-east India. We selected ten tree species (3 mammal-dispersed and 7 bird-dispersed) that varied in seed size and followed the fates of 10,777 tagged seeds. We used camera traps to determine the identity of rodent visitors, visitation rates and their seed-handling behavior. Seeds of all tree species were handled by at least one rodent taxon. Overall rates of seed removal (44.5%) were much higher than direct on-site seed predation (9.9%), but seed-handling behavior differed between the terrestrial rodent groups: two species of murid rodents removed and cached seeds, and two species of porcupines were on-site seed predators. In addition, a true cricket, *Brachytrupes* sp., cached seeds of three species underground. We found 309 caches formed by the rodents and the cricket; most were single-seeded (79%) and seeds were moved up to 19 m. Over 40% of seeds were re-cached from primary cache locations, while about 12% germinated in the primary caches. Seed removal rates varied widely amongst tree species, from 3% in *Beilschmiedia assamica* to 97% in *Actinodaphne obovata*. Seed predation was observed in nine species. *Chisocheton cumingianus* (57%) and *Prunus ceylanica* (25%) had moderate levels of seed predation while the remaining species had less than 10% seed predation. We hypothesized that seed traits that provide information on resource quantity would influence rodent choice of a seed, while traits that determine resource accessibility would influence whether seeds are removed or eaten. Removal rates significantly decreased (*p* < 0.001) while predation rates increased (*p* = 0.06) with seed size. Removal rates were significantly lower for soft seeds (*p* = 0.002), whereas predation rates were significantly higher on soft seeds (*p* = 0.01). Our results show that murid rodents play a very important role in affecting the seed fates of tropical trees in the Eastern Himalayas. We also found that the different rodent groups differed in their seed handling behavior and responses to changes in seed characteristics.

## Introduction

Spatial distribution of seeds in a forest is determined by primary dispersal agents and by post-dispersal events such as seed predation. After primary dispersal by fruit-eating animals, the seeds may be transported further by other consumers [1]. Apart from ants and dung beetles, terrestrial rodents are most responsible for secondary seed dispersal [2, 3, 4]. In temperate, single-species dominant conifer forests with pronounced seasonality, tree squirrels and chipmunks are known to larder-hoard seeds for consumption during resource-poor winter months [1, 5, 6]. Scatter-hoarding, where seeds are stored in several scattered caches (mostly single seed per cache) resulting in low spatial density of seeds, is more prevalent in tropical forests [7]. Rodents move seeds towards sites of lower conspecific tree densities and bury them, which provides suitable conditions for germination [8, 9]. During this multi-step pathway, rodents cache more seeds than they can manage to retrieve, and some seeds may eventually escape being eaten and have a higher probability of establishing into seedlings [10, 11, 12, 13]. This phenomenon tends towards a mutualistic relationship between plants and their rodent dispersers, as rodents depend on their stored reserves during periods of resource shortage and plants depend on rodents for dispersal [14].

Most studies on scatter-hoarding rodents (mainly agoutis *Dasyprocta* sp. and acouchies *Myoprocta* sp.) and their interactions with tree species are from the Neotropics [7, 13, 15, 16, 17, 18, 19]. There has been relatively little research on the role of rodents in Asian forests, although the region contains a high diversity of terrestrial (Muridae, Hystricidae) and arboreal pre- and post-dispersal seed predators (Sciuridae). Recent studies have documented scatter-hoarding by terrestrial rodents such as the nocturnal *Leopoldamys sabanus*, *Maxomys* spp., and *Menetes berdmorei* along with diurnal squirrels such as *Lariscus insignus* and *Callosciurus finlaysonii* from Malaysia and Thailand [20, 21, 22]. Detailed studies from subtropical forests in China report scatter-hoarding by terrestrial rodents such as *Leopoldamys edwardsi* and by species belonging to the genera—*Rattus*, *Niviventer*[23, 24, 25, 26, 27]. Seed predation by other rodents such as *Hystrix brachyura* has been reported in some studies [27, 28].

A recent study in north-east India monitored the effect of terrestrial rodents on five hornbill-dispersed tree species and reported high seed predation (73%) and low rates of secondary dispersal (1.4%) during the drier months of the year [29]. However, there is potential for seasonal and inter-annual variation in rates of seed predation and seed caching by rodents as their populations fluctuate and resource abundance varies. More studies on secondary dispersal and seed predation by rodents on a wider range of tree species within a plant community throughout the year are necessary to understand the prevalence and importance of these mechanisms in a forest. There is also a need to understand different mechanisms that influence seed fates in Asian forests, as the various rodent groups here belong to different genera and families and are very different morphologically from the Neotropical rodents that are well-documented secondary seed dispersers [13, 15].

In this study, we aimed to identify the rodent seed predators and secondary seed dispersers in a tropical forest in north-east India, and track seed fates of ten tree species to understand the prevalence of seed caching and cache characteristics. Forget *et al*. [30] have shown that caching (by large Neotropical rodents) is greater during periods of declining fruit abundance than during periods of either high or low fruit abundance in the Neotropics. In our study site in Asia, the relatively small-bodied murid rodents are expected to scatter-hoard seeds and are likely to display population fluctuations and therefore, seasonal changes in scatter-hoarding rates. Although the extent of seasonal fluctuations in porcupines, which are known seed predators, is unknown, given their larger body size, they may have more stable populations across seasons thereby maintaining predation rates. Apart from season, seed traits such as seed size and mechanical or chemical defenses against predation are important for influencing post-dispersal seed fates [20, 31, 32]. In the Neotropics, large seeds are removed faster and to farther distances than smaller seeds as they provide more resources to the rodents [11, 31]. However, studies from the Paleotropics have shown that seeds of intermediate sizes are preferred by the rodents for removal and caching, which is likely due to the smaller-sized rodents in the region [32, 33]. Also, seeds that have natural defenses against predators have lower rates of predation and a higher chance of being scatter-hoarded [20, 34].

Based on the earlier studies and given that the rodent community in our study site includes both murid and hystricid rodents that vary in their body size, we expected to find differences in their seed handling behavior. We hypothesized that seed traits that provide information on resource quantity (such as seed size) should influence rodent choice or selection of a seed, whereas, those that determine resource accessibility (eg. mechanical barriers to predation) should influence their seed handling behavior(i.e. whether they are removed or eaten). We tested the above hypothesis with the following predictions: (1) Rodents are expected to choose larger seeds over smaller ones, however such selection may be constrained by their own body sizes; (2) An increase in seed coat thickness is expected to decrease immediate on-site predation and increase removal rates as harder seeds can be stored for a longer time (less prone to fungal infestation and longer dormancy/germination times). We report variation in seed fates among tree species due to variation in seed traits and differences in seed handling behaviors among rodent species.

## Material and Methods

### Ethics statement

A research permit to conduct this study was obtained from the Arunachal Pradesh Forest Department. This study did not involve any animal handling.

### Study area

**Fig 1:**
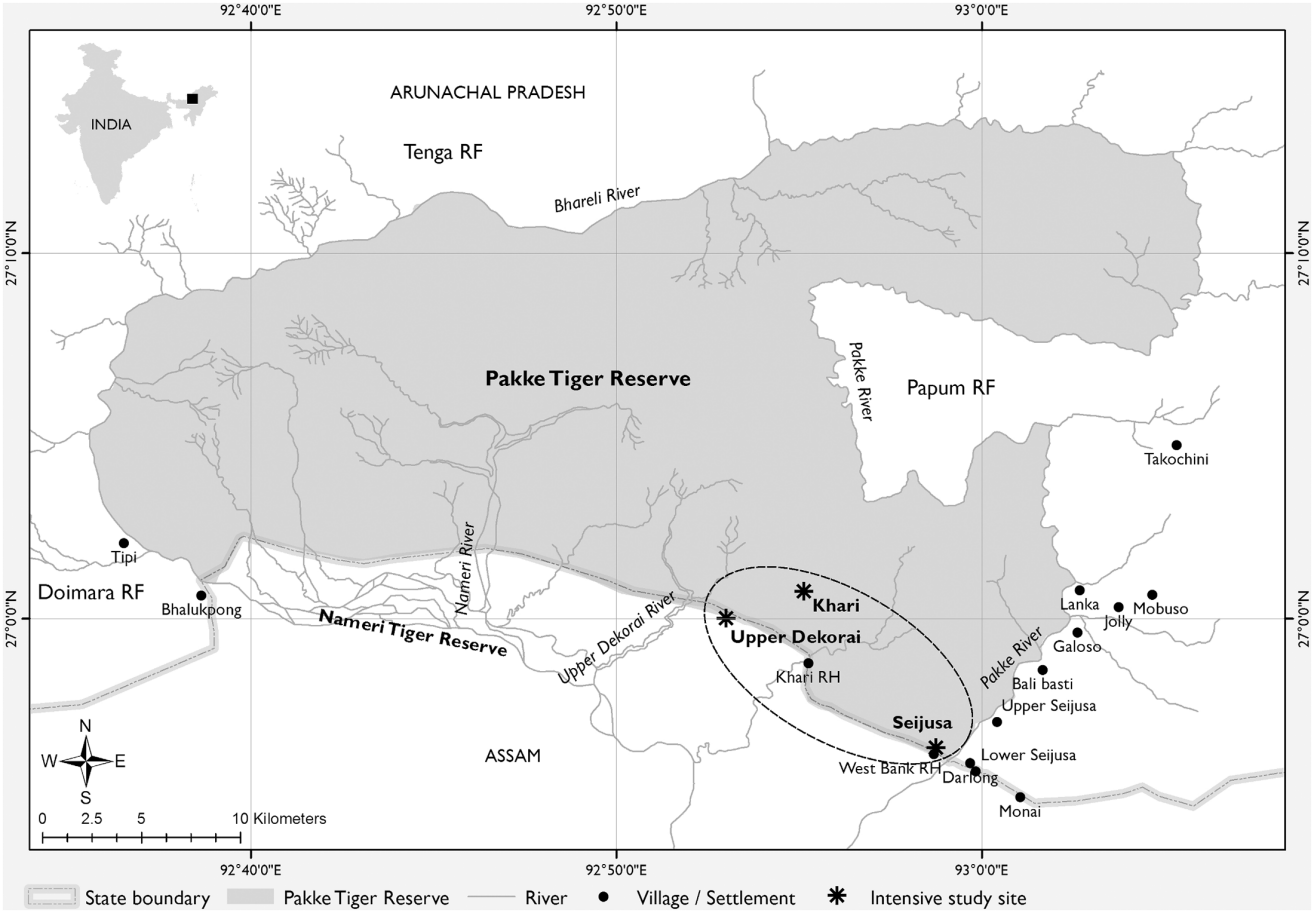
A map of the study area, Pakke Tiger Reserve, located in the Eastern Himalaya, India. The intensive study sites (shown as three points inside the ellipse) were situated in the south-eastern part of the Reserve.

The study was conducted in Pakke Tiger Reserve (26°54´–27°16´N, 92°36´–93°09´E; 862 km^2^), a tropical forest in the Himalayan foothills in the state of Arunachal Pradesh, India (Fig 1). Our study region is part of the Indo-Burma global biodiversity hotspot [35, 36]. The main vegetation type is categorized as semi-evergreen and moist deciduous forest [37]. This area receives an annual rainfall of 2500 mm, most of which falls during the south-west monsoon (June to September) and has an altitudinal range of 150 to 2000 m asl. Some of the dominant tree species in the study site include: *Polyalthia simiarum*, *Pterospermum acerifolium*, *Sterculia alata*, *Stereospermum chelonoides*, *Ailanthus grandis*, *Duabanga grandiflora* [38]. Prior to its declaration as a protected area (Game Sanctuary) in 1977, a small area in the south-eastern part had experienced selective logging [39]. Hunting by indigenous tribes that used traditional trapping devices and firearms was also prevalent in the area. In 2002, it was declared a Tiger Reserve giving it a higher level of protection. Even after establishment of the protected area, occasional hunting was prevalent. However in the last decade, stricter enforcement and protection by the Forest Department has ensured that hunting inside the reserve has stopped [40].

We collected field data from June 2011 through June 2012 in an intensively sampled study area that was located in the south-eastern part of the reserve and was spread out between three sites: Seijusa (26°56´N-92°58´E), Upper Dekorai (27°0´N-92°52´E), and Khari (27°0´N-92°55´E) (Fig 1). The altitude in this area ranges from 150 to 220 m asl. The southern edge of the study area lies close to an inter-state boundary with Assam and has witnessed occasional cane extraction by local people [39]. The Pakke river provides a natural boundary on the eastern edge of the reserve. The habitat type near the south-eastern edge is mainly secondary semi-evergreen forest which changes into mature primary forest towards the interior.

The study area is likely to harbour about 20 species of terrestrial rodents (10 to 2500 g). The rodent community includes two species of porcupines, Himalayan crestless porcupine *Hystrix brachyura* and Asiatic Brush-tailed porcupine *Atherurus macrourus*, both of which are known seed predators [29]. Other terrestrial rodents specifically reported from the area are murid rodentssuch as *Rattus* sp., *Berylmys* sp., *Bandicota* sp., *Niviventer* sp.[29], and potentially the Edwards's long-tailed rat *Leopoldamys edwardsi*. These murid rodent species (100–400 g) are known to scatter-hoard in China [24, 27]. Four species of primarily arboreal diurnal squirrels that are pre-dispersal seed predators include the Himalayan striped squirrel *Tamiops macclellandi*, hoary-bellied squirrel *Callosciurus pygerythrus*, Pallas red-bellied squirrel *Callosciurus erythraeus*, and Malayan giant squirrel *Ratufa bicolor* [41].

### Sampling methods

#### Study species

We selected 10 tree species that fruit in different seasons. Tree species fruited across the entire study period which was categorized into three different seasons: wet (May to August), dry (November to February), and intermediate (September to October and March to April) (Table 1). The tree species were also selected based on a variation in seed size and dispersal modes (Table 1). Seed sizes ranged from ≤ 5 mm (2 species) to 40 mm, with two mammal-dispersed tree species, four bird-dispersed tree species and four tree species that were dispersed by both birds and mammals (Table 1).

**Table 1:** Study species and their seed fates. Seed fates of ten study tree species observed inside experimental seed plots during June 2011–June 2012. Each species was monitored for a period of 50 days from the onset of peak fruiting of each tree species. The tree family and dominant dispersal mode for each species are presented (Source: Datta and Rawat 2008 [38], Velho *et al*. 2012 [49], Datta 2001 [50], Sethi & Howe 2012 [64]).

Species	Family	Dispersal mode	Fruiting months	Seed size(mm)	Seed coat	Seeds sampled	Removed (%)	Preyed upon (%)	Alive (%)	Dead (%)	Germinated (%)
*Chisocheton cumingianus*	Meliaceae	Bird	May	30	Soft	1002	39.3	56.6	0	4.1	0
*Elaeocarpus aristatus*	Elaeocarpaceae	Mammal	June–July	18	Hard	1061	51.8	0.3	36.9	11	0
*Turpinia pomifera*	Staphylaceae	Mammal	July–August	5	Hard	1325	58.3	0	40.8	0.9	0
*Litsea sp*.	Lauraceae	Bird, mammal	July–August	17	Soft	1069	17.7	0.3	0.1	9.4	72.6
*Actinodaphne obovata*	Lauraceae	Bird	September–October	17	Soft	883	96.8	0.7	1.2	1.2	0
*Talauma hodgsonii*	Magnoliaceae	Bird, mammal	October–November	4	Hard	1125	94	6	0	0	0
*Horsfieldia kingii*	Myristicaceae	Bird	February–March	36	Soft	1084	61.2	8.1	8.4	22.3	0
*Beilschmiedia assamica*	Lauraceae	Bird	November	31	Soft	1020	2.8	2.5	61.5	33.2	0
*Canarium resiniferum*	Burseraceae	Bird, mammal	December–January	40	Hard	1121	8.2	3.2	88.6	0	0
*Prunus ceylanica*	Rosaceae	Bird, mammal	January-February	22	Soft	1087	17.5	25.1	52.9	4.5	0
Overall seed fates	-	-	-	-	-	10,777	44.5	9.9	30	8.5	7.2

We gathered ripe fruits to obtain seeds of all study tree species during their peak fruiting period from under parent fruiting trees. We removed fruit pulp by rubbing the fruits with a clean dry cloth and then air-drying the seeds for 2–4 hours. Following Velho *et al*. [28], we expected rodents to be searching for seeds randomly in the forest, but we also expected rodent activity to be high near the parent fruiting tree, a known source of seeds to rodents. With this in mind, seeds were laid out following two different sampling strategies: around parent fruiting trees, and away from parent fruiting trees in a randomly selected location inside the forest. For sampling near parent trees, we selected ten fruiting tree individuals for all study speciesexcept for *A*. *obovata* and *H*. *kingii* where five and nine individuals were chosen, respectively. *A*. *obovata* and *H*. *kingii* are both dioecious species, and we were able to locate relatively fewer fruiting female individuals of these species. *H*. *kingii* occurs at relatively low densities (1.1 tree/ha), while *A*. *obovata* occurs at a density of 3.2 trees/ha. Seed plots were laid out at two distances radiating from the tree trunk. There were two plots closer to the parent tree (located at the crown edge of individual trees) and two plots were placed 20 m away from the centre of the tree. Therefore, for all species we had 40 plots, except for *A*. *obovata* and *H*. *kingii* where we had 20 and 36 plots, respectively. All conspecific tree individuals were spaced at a minimum distance of 30 m and the seed plots radiated away from the direction of their nearest neighbour. A total of 376 plots were sampled around all selected tree individuals (94 trees). We placed 10 seeds/plot for 81 trees. Two *A*. *obovata* trees and one *E*. *aristatus* trees bore fewer fruits, and therefore due to non-availability of sufficient seeds, we placed 6 seeds/plot for these three individuals. In addition, due to the small size of *T*. *pomifera* seeds, combined with a large fruit crop, we placed 15 seeds/plot for all ten individuals of this species.

In addition to the plots near parent trees, we laid out a series of 15 plots. All seed plot experiments were conducted during the peak fruiting period of each tree species between June 2011 and June 2012 (Table 1, S1 Table). The plots were placed along a transect line with a spacing of 50 m between each plot. These plots were at least 50 m away from conspecific fruiting trees and contained a random number of seeds between 1 and 100 that were generated using 'RAND' function in OpenOffice version 3.3, and represented a density gradient of seeds (following Velho *et al*. [42]).

We cleared leaf litter on the forest floor to mark all 2×2 m^2^ seed plots that were placed either near or away from parent fruiting trees. Seeds within these plots were tagged using 50 cm long Dacron fishing line which was attached to the seed surface using Loctite Super Glue. Seeds of *T*. *hodgsonii and T*. *pomifera* were not tagged as they were too small. We also marked each tagged seed with a permanent marker.

#### Camera trap monitoring

We carried out camera trap monitoring on seed plots during August 2011 –June 2012. The aim was to establish the identity of the main seed predators and caching species, observe their seed handling behavior which is defined as either seed removal or seed predation, and determine visitation rates and the fates of seeds that were handled by the visitors. We had a limited number of camera traps and set out 10 Reconyx HC500 camera traps at 88 out of 526 seed plots of seven study species. Each camera trap was triggered when an animal entered its range or field of view and intercepted an infra-red beam emitted from the camera trap, whereupon the camera trap recorded 3 pictures per second while the animal remained in range. The duration for which an animal remained continuously in view of the camera was defined as one visit. We aimed to observe each tree species for a minimum of 50 trap nights. However, for one species (*Actinodaphne obovata*) rodents removed seeds within 44 trap nights. For the other six species, we had an effort > 50 trap nights (63–230).

#### Monitoring seed fate

The fate of each tagged seed was monitored every 3^rd^ day for up to 30 days and subsequently once a week up to 50 days or until time of germination or complete removal/predation, whichever was first. The following seed fates were noted for all individual seeds: (i) alive or viable, (ii) preyed upon or dead due to predation by rodents, (iii) dead due to other causes (insects, fungal attack), (iv) germinated, (v) removed, (vi) cached. When seeds were 'preyed upon' by rodents, the seed remains and tags were found inside seed plots which helped us confirm the seed fate. Seeds that were categorized as 'removed' included the seeds that were not found inside a plot during a monitoring session. Whenever seeds were absent from the plot, three people searched in a minimum 10 m radius around the plot to locate the ‘removed’ seeds. When a removed seed was found it was termed as ‘cached’ and the location of the seed was considered to be a ‘primary’ cache. For all cached seeds, we noted cache substrate (if the cache was under leaf-litter, inside tree hollow or underground) and the distance of the cached seeds from the seed plot. During every monitoring session at a cache, we noted the number of seeds and fate of seeds in the cache (alive, preyed upon, dead, germinated, and removed).

It is possible that some of these ‘removed’ seeds may have been moved to a safer location for consumption by the rodents, and not for caching. We carried out an additional experiment in February2014 for a period of 15 days to see if clearing of leaf litter from the plot influenced rodent behavior due to associated increase in the risk of predation. We used a paired plot design where we cleared leaf-litter in one plot but not in the other. Five replicates each with 20 seeds per plot of *P*. *ceylanica* were used for cleared and uncleared plots. We did not find any signs of on-site predation in any of the plots. One-way ANOVA test results did not show a significant difference in the number of seeds removed between cleared and uncleared plots (*F*_*1*,*8*_ = 2.35, *df* = 1, *p* = 0.16). This suggests that the clearing of the plots did not affect the behavior of the rodents.

### Data analysis

We calculated the percentage of seeds under various seed fate categories for different tree species. Camera trap data were used to quantify the number of visits by different animal species, number of seeds handled per visit, and fate of seeds being monitored such as on-site seed predation or removal away from the seed plot. We calculated average number of visitations and percentage of seeds handled by visitors. We pooled the data collected from plots located near to and away from the parent trees of each species to calculate the percentage of seeds handled by different rodent species. However, for reporting the average visitation rates (number of visits per trap-night) on seed plots, we used data only from far away plots, as this was the larger dataset.

We used a parametric approach to explore the effect of seed size, and seed-coat thickness on seed removal rates (proportion of seeds removed) and seed predation rates (proportion of seeds preyed upon). Seed size was used as a continuous variable and ranged from 4.0 mm to 40.4 mm. Seed-coat thickness was defined as either "soft" (when the seed could be cut using a sharp kitchen knife) or "hard" (when it could not) (Table 1). To test our predictions, we developed Generalized Linear Models (GLM). We included proportion of seeds removed (model 1) and preyed upon (model 2) as response variables in the GLM, and seed size and seed-coat thickness as explanatory variables. Seed size was included as a continuous variable and seed-coat thickness as a categorical variable. Seed density in experimental plots which was expected to influence rates of seed removal and predation was not included in the final models as it did not affect proportion of seeds removed (z-value = 581, *df* = 239, *p* = 0.561) or proportion of seeds preyed upon by rodents (z-value = -0.285, *df* = 239, *p* = 0.776). Similarly, the location of plots (near and far from parent trees) did not influence either seed removal rate (z-value = 0.824, *df* = 238, *p* = 0.410) or predation rate (z-value = -1.193, *df* = 238, *p* = 0.233), and therefore, was not included in the final analyses. Data from plots that were located near to and far from parent tress were pooled.

We used a 'binomial' error family and 'logit' link function in the model as our response variable data were in proportions [43]. Lower values of residual deviance (155 and 96) in comparison to residual degrees of freedom (239 and 240) for model 1 and 2, respectively, suggested that the data were not over-dispersed [44]. However, the ratios of the residual deviance to the residual degrees of freedom suggested under-dispersion (< 1). The problem of under-dispersion is usually addressed by carrying out a quasibinomial GLM, instead of a binomial GLM (Ben Bolker, *pers*. *comm*.). We carried out a quasibinomial GLM, where the variance is lower than expected from a binomial process. For such cases, the GLM includes 'quasi' exponential functions that add the parameter phi (φ) to the expected variance equation. The expectation is that the fit of φ will be greater than 1 if data is over-dispersed and <1 if it is under-dispersed). The 'quasibinomial' family is used for binomial data in R. However, the results from the quasibinomial indicated under-dispersion for model 1 (seed removal), while indicating over-dispersion for model 2 (seed predation). This was in contradiction with the estimate of dispersion based on the ratio of residual deviance to residual degrees of freedom. Another estimator of dispersion, the Pearson’s Chi-square indicated over-dispersion for model 2. Given the conflicting and different estimates of dispersion for the response variable, we used bootstrapping to generate confidence intervals around the parameter estimates to address the problem (Ben Bolker, *pers*. *comm*.).

Season is an important factor that is likely to affect rates of predation and removal. However, season was not included in the GLM models as different species produce fruit during different seasons and it is difficult to tease apart the effect of species from the effect of season. Three different seasons were defined: wet (May to August), dry (November to February), and intermediate (September to October and March to April) (Table 1). Using non-parametric approaches, we visually explored the effect of season and seed-coat thickness on seed removal and seed predation rates; boxplots were created using season and seed-coat thickness as categorical variables. To visually explore the effect of seed size, scatter-plots were created with Locally Weighted Scatterplot Smoothening (LOWESS) using seed size as a continuous variable.

We used the data on cached seeds to explore cache characteristics such as distance of cache from original location of seed station and number of seeds per cache. We also calculated the proportion of seeds found under various seed fates in the caches made by different animals as well as in different substrate types such as under leaf-litter, and inside tree hollow.

We used R statistical and programming environment, Version 3.0.2, for all statistical analyses [45].

## Results

### Seed removal and on-site seed predation

We monitored 10,777 seeds belonging to ten tree species in 526 seed plots and observed that 4,793 (44.5%) seeds were removed and 1,068 (9.9%) seeds were preyed on-site by vertebrates (Table 1). Out of the 4,793 tagged seeds that were removed, we located 489 seeds (10%) to primary caches. The remaining 4,304 seeds could not be tracked post-removal as the animals often cut off the tags before removing the seeds. Of the remaining 4,916 seeds that were not removed or preyed upon, 3,229 (30%) seeds were alive, 911 (8.5%) died due to fungal/insect infestation, and 776 (7.2%) germinated by the end of experiments. All the germinating seeds inside seed plots belonged to *Litsea* sp. (Table 1).

*C*. *resiniferum* and *B*. *assamica* had the least number of seeds handled by rodents, 11.4% and 5.3%, respectively, while *T*. *hodgsonii*, *C*. *cumingianus* and *A*. *obovata* had over 90% of their seeds either removed or preyed on (Table 1). The proportion of seeds removed per tree species ranged from 2.8% for *B*. *assamica* to 96.8% for *A*. *obovata*, while observed on-site predation ranged from zero for *T*. *pomifera* to 56.6% for *C*. *cumingianus* suggesting differences in rodent food choice.

### Identity of on-site seed predators, seed hoarders and visitors

We obtained 1,035 rodent visits during 26,569 photographic captures in 1,082 camera trapnightsand monitored 2,735 (25.5% of all seeds sampled) seeds in 88 plots (16.7% of all plots) (S2 Table). Based on the above data, we found that the seeds were handled by two species of murid rodents (S1 Video) belonging to *Niviventer* and *Rattus* species, one species of squirrel (hoary-bellied squirrel, S2 Video), and two species of porcupines (Himalayan crestless porcupine and Asiatic brush-tailed porcupine, S3 Video).

The on-site seed predators were Himalayan crestless porcupine, brush-tailed porcupine, and the hoary-bellied squirrel. We recorded evidence for predation on 433 (15.8%) out of 2,735 seeds through our camera trap photographs.

Camera trap photographs revealed that 828 (30.3%) of 2,735 seeds being monitored were removed or carried away from plots by murid rodents and the hoary-bellied squirrel. These records, together with direct observations on 489 cached seeds that we were able to locate after removal from our experimental plots, provide evidence that secondary seed dispersal by murid rodents and hoary-bellied squirrel occurs in our study area.

We also found that a species of true cricket *Brachytrupes* sp. (S1 Image) removed and cached seeds underground during the rainy season (May–August), usually 10–25 cm below the soil surface. The seeds cached by the cricket were buried, while the tags remained partially outside on the surface, which enabled us to locate these seeds. At the end of the monitoring period, we dug out the underground caches to examine their fates and found four dead individuals of this cricket species inside the burrows along with the seeds. Several seeds revealed feeding marks, most likely made by the action of mandibles of insects and not by a vertebrate animal. Our camera traps were not able to capture the insect which is likely due to its relatively small size (head to abdominal tip: 4.8 cm). Therefore, based on this evidence, we infer that the seeds of *C*. *cumingianus*, *Litsea* sp., and *E*. *aristatus* were also removed and cached by the cricket species. Among other visitors, emerald dove (*Chalcophaps indica*) was the only species that was observed handling seeds.

Other mammal species that visited the plots but did not handle or consume seeds were the small Indian civet (*Viverricula indica*), large Indian civet (*Viverra zibetha*), crab-eating mongoose (*Herpestes urva*), leopard cat (*Prionailurus bengalensis*), marbled cat (*Pardofelis marmorata*), wild dog (*Cuon alpines*), Indian muntjac (*Muntiacus muntjak*), Asian elephant (*Elephas maximus*), Northern tree shrew (*Tupaia belangeri*) and rhesus macaque (*Macaca mulatta*). Other avian visitors included grey-headed woodpecker (*Picus canus*), orange-headed thrush (*Zoothera citrina*), Himalayan whistling thrush (*Myophonus caeruleus*), white-rumped shama (*Copsychus malabaricus*), white-throated fantail (*Rhipidura albicollis*), red jungle fowl (*Gallus gallus*), grey peacock-pheasant (*Polyplectron bicalcaratum*), white-cheeked partridge (*Arborophila atrogularis*), and the hooded pitta (*Pitta sordida*).

### Visitation, seed removal and seed predation rates

**Fig 2:**
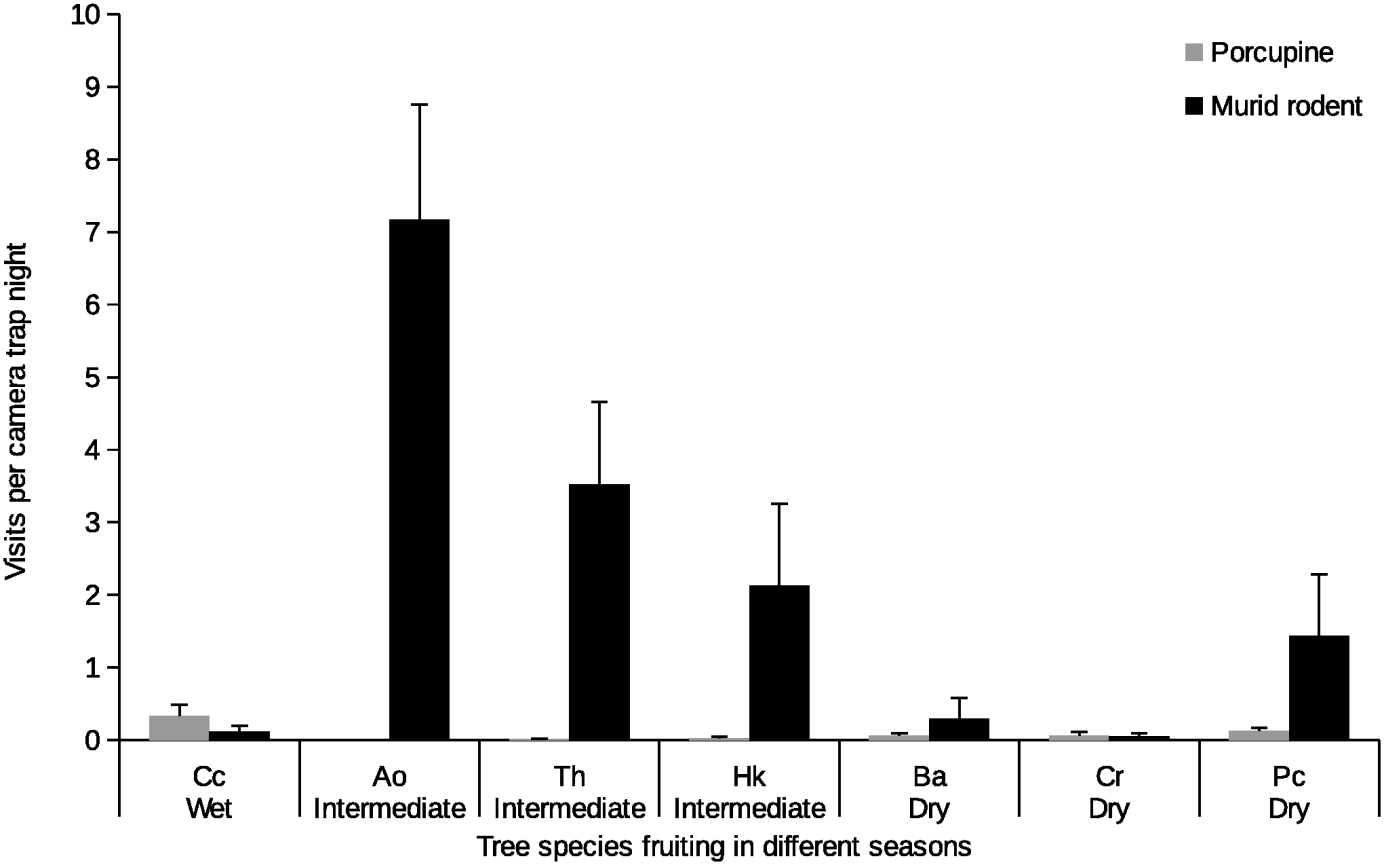
Rodent visitation rates on seed plots. Average number of visitations (± SE) by terrestrial rodents per camera trap night on experimental plots located in random forest sites for seven study tree species in different seasons. Sampling effort in terms of camera trap nights, total number of seeds sampled per species and total number of seed plots for tree species is: Ao–*Actinodaphne obovata* (8, 70, 3), Cc–*Chisocheton cumingianus* (58, 288, 6), Th–*Talauma hodgsonii* (58, 503, 7), Hk–*Horsfieldia kingii* (166, 386, 6), Pc–*Prunus ceylanica* (126, 299, 4), Cr–*Canarium resiniferum* (94, 501, 7), Ba–*Beilschmiedia assamica* (30, 183, 3).

**Fig 3:**
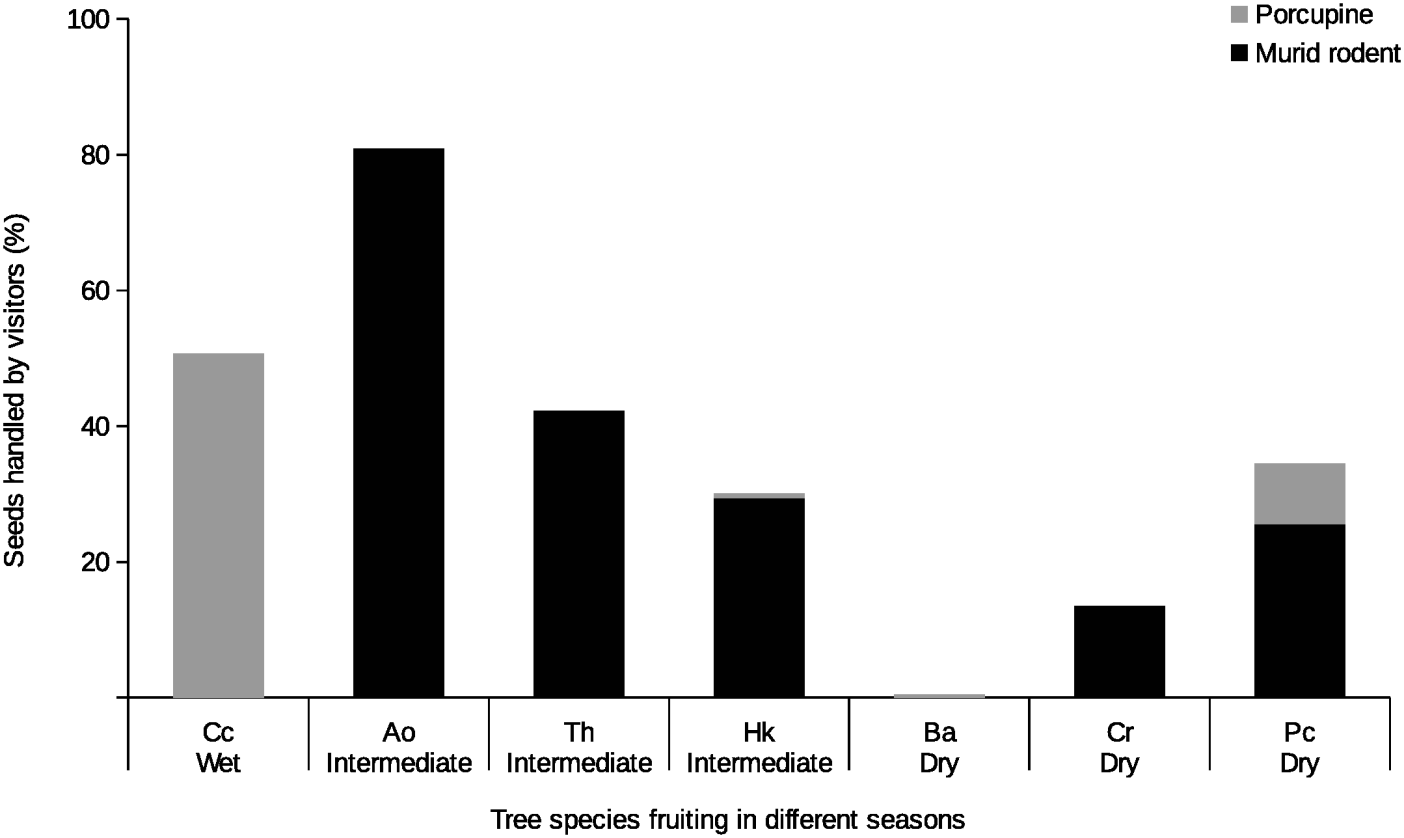
Seed handling by rodents. Percentage of seeds handled (out of total tagged seeds) by murid rodents and porcupines in seed plots for seven study tree species in different seasons. All the seeds handled by murid rodents were removed whereas all the seeds handled by porcupines were preyed upon. Sampling effort in terms of camera trap nights, number of seeds sampled per species and number of total seed plots for tree species is: Ao–*Actinodaphne obovata* (44, 146, 11), Cc–*Chisocheton cumingianus* (188, 374, 15), Th–*Talauma hodgsonii* (63, 533, 9), Hk–*Horsfieldia kingii* (215, 460, 14), Pc–*Prunus ceylanica* (230, 396, 14), Cr–*Canarium resiniferum* (156, 527, 10), Ba–*Beilschmiedia assamicus* (186, 299, 15).

A total of 79 out of 88 plots with camera traps were discovered by rodents. Among terrestrial rodents, murid rodents were generally the most frequent visitors and handled the highest proportion of seeds by always removing them from seed plots (Figs 2 and 3). Murid rodents visited seed plots of tree species that fruited in the intermediate months (between wet and dry seasons) more frequently (Fig 2). The experimental plots that were most visited by murid rodents belonged to *A*. *obovata*, *T*. *hodgsonii*, and *H*. *kingii*, with seed plots of *A*. *obovata* undergoing 81% seed removal, the highest among all the species sampled (Fig 3).

The visitation rates to seed plots by porcupines followed those of murid rodents. Their seed handling behavior differed from that of murids as porcupines were always on-site seed predators, and were never observed to carry away seeds. *C*. *cumingianus* was observed to undergo the highest rate of seed predation by porcupines (Fig 3).

An arboreal rodent species, the hoary-bellied squirrel visited seed plots of only two species, *C*. *cumingianus* and *T*. *hodgsonii*; the average visitation rates (visits per trap night) by the squirrel to *C*. *cumingianus* were 1.34 (± 1.10 SE) and to *T*. *hodgsonii* were 0.33 (± 0.21 SE). While *T*. *hodgsonii* was preyed upon by the squirrel, *C*. *cumingianus* seeds were scatter-hoarded among leaf-litter where they had potential for germination.

Of all the bird visitors, only the emerald dove was observed handling seeds. This bird species preyed upon the seeds of *T*. *hodgsonii*, consuming 57 of 503 seeds of this species in a single visit at one camera-trapped seed plot.

### Factors affecting community-level seed fate patterns

The GLM results showed that increased seed-coat thickness affected the removal of small seeds more than large seeds (Table 2). At smaller seed sizes, hard seeds were removed more than soft seeds, but as seed size increased, seed-coat thickness was no longer important for seed removal. Predation rates were not significantly affected by seed size (*p* = 0.06) but significantly affected by seed coat (*p* = 0.01). The interaction term between seed size and seed coat was not significant for predation and therefore, removed from the analysis. Predation rates were higher on soft seeds than on hard seeds.

**Table 2:** Effect of seed traits on seed removal and seed predation. Coefficients of a quasibinomial generalized linear models (GLM) for: (a) proportion of seeds removed with seed size (as a continuous variable), seed-coat thickness(soft or hard), and an interaction between seed size and seed coat; and (b) proportion of seeds preyed upon with seed size and seed coat. The coefficient estimates of proportion of seeds removed and preyed upon are presented on a logit link scale as log odds values. The sign ('+' or '-') of coefficient estimates indicates the relationship among variables. The bootstrapping (10000 runs) generated confidence intervals (CI) around the parameter estimates are also presented here along with the associated *p*-values (*p*_*b*_).

Variable	Coefficient estimate	*df*	*Z-value*	*p*	Lower CI	Upper CI	*p*_*b*_
*(a) Proportion of seeds removed*							
Intercept	1.709	242	4.501	<0.001	1.281	2.23	<0.001
Seed size	-0.098	241	-4.763	<0.001	-0.125	-0.079	<0.001
Seed coat (soft)	-1.687	240	-2.245	0.002	-2.932	-0.469	<0.01
Seed size x seed coat	0.074	239	2.302	0.002	0.024	0.124	<0.005
*(b) Proportion of seeds preyed upon*							
Intercept	-5.509	242	-4.647	<0.001	-10.442	-3.603	<0.001
Seed size	0.058	241	2.032	0.06	0.016	0.115	<0.005
Seed coat (soft)	2.342	240	2.762	0.01	1.267	6.349	<0.001

**Fig 4:**
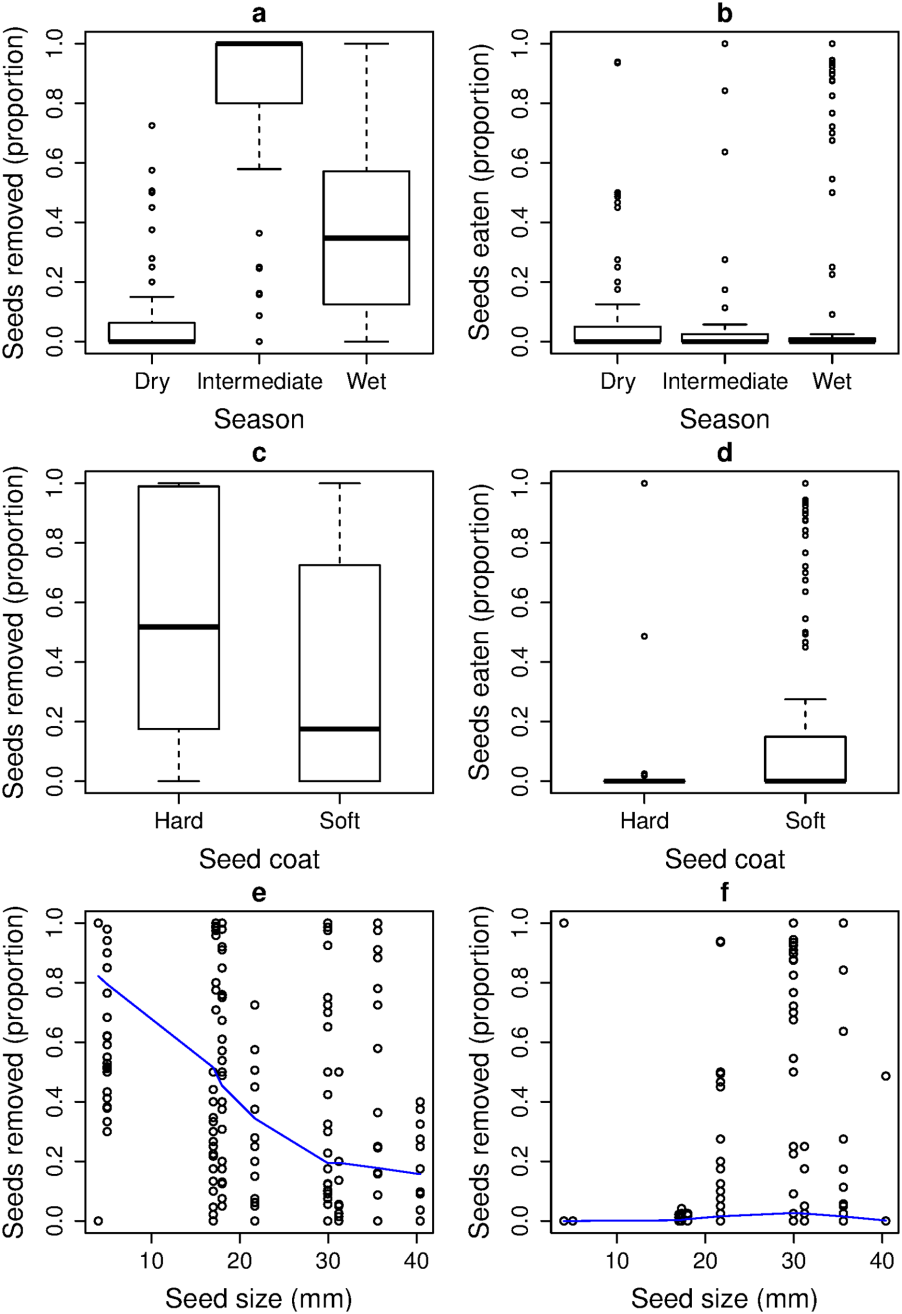
Patterns in seed handling by rodents. Relationship between proportion of seeds removed and proportion of seeds eaten or preyed upon with respect to season (a, b), and seed coat (c, d) using boxplots (median with the interquartile range and the whiskers representing data ranges) and with respect to seed size (e, f) using scatterplots and fitting the line with locally weighted scatterplot smoothing (LOWESS).

We visually explored the effect of season, which was not modelled in the GLMs, and found that the seed removal rates appeared to be higher in the intermediate season than either wet or dry season whereas seed predation rates were low in all the seasons (Fig 4a and 4b). Removal rates tended to increase from soft seeds to hard seeds, whereas predation rates showed an opposite trend (Fig 4c and 4d). Removal rates decreased with increase in seed size, while seed predation rates remained low, irrespective of seed size (Fig 4e and 4f).

### Seed caching, cache characteristics and seed fate in primary caches

We located 489 of our tagged seeds in 309 caches. Murid rodents cached 280 seeds (57%), followed by 147 (30.3%) seeds cached by the true cricket *Brachytrupes* sp., and 62 seeds (12.6%) cached by the hoary-bellied squirrel (Table 3).

**Table 3:** Seed fates of cached seeds. Seed fates of the seeds inside the caches of different cache owners. N = 489 cached seeds.

Animal species (cache owner)	Removed (%)	Preyed upon (%)	Alive (%)	Dead (%)	Germinated (%)
True cricket *Brachytrupes* sp. (n = 147)	57.8	2.04	14.29	17.01	8.84
Murid rodent (n = 280)	48.21	41.43	7.14	2.86	0.36
Hoary-bellied Squirrel *Callosciurus pygerythrus* (n = 62)	93.55	0	1.61	3.23	1.61

The cache size ranged from a single seed to a maximum of 13 seeds. Most caches were of single seeds (78.6%, n = 243), followed by caches of two seeds (9.7%, n = 30). Average size of caches made by cricket (n = 71), murid rodent (n = 176), and squirrel (n = 62) were 2.1 (± 0.2 SE) seeds, 1.6 (± 0.2 SE) seeds, and 1 (± 0 SE) seed, respectively.

**Fig 5:**
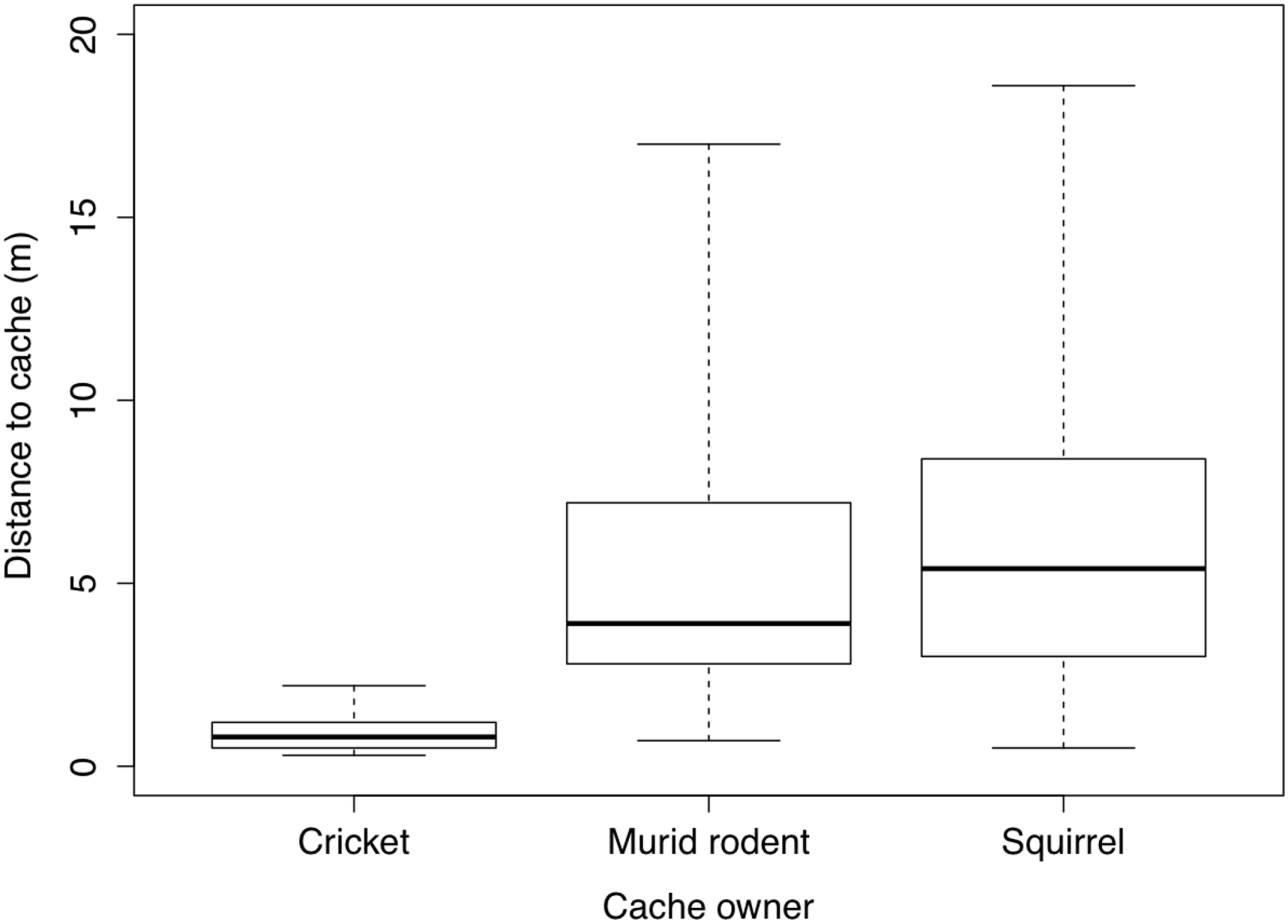
Distance to primary cache locations. A boxplot representing distances (in metres) between experimental plots and primary caches made by different cache owners: cricket (*Brachytrupes* sp., n = 71 caches), murid rodent (n = 176 caches), and squirrel (hoary-bellied squirrel, n = 62 caches).

The average distance that a seed was moved between a seed plot and a primary cache was 0.88 m (range = 0.3–2.2 m, n = 147) by the crickets, 5.3 m (range = 0.7–17 m, n = 280) by murid rodents, and 6.2 m (range = 0.5–18.6 m, n = 62) by the hoary-bellied squirrel (Fig 5). All the caches made by the cricket species were underground, usually below 10 cm and up to 25 cm. In some of these caches, we observed that the seeds were dug out, and re-buried. Squirrel caches were made by burying seeds under leaf-litter and sometimes in topsoil.

Murid rodents cached 280 seeds in a diverse manner. A majority of seeds were cached under leaf litter (n = 202), followed by several seeds inside alive or dead tree hollows (n = 74). The remaining seeds were cached either inside buttresses, or under fallen logs and snags.

**Fig 6:**
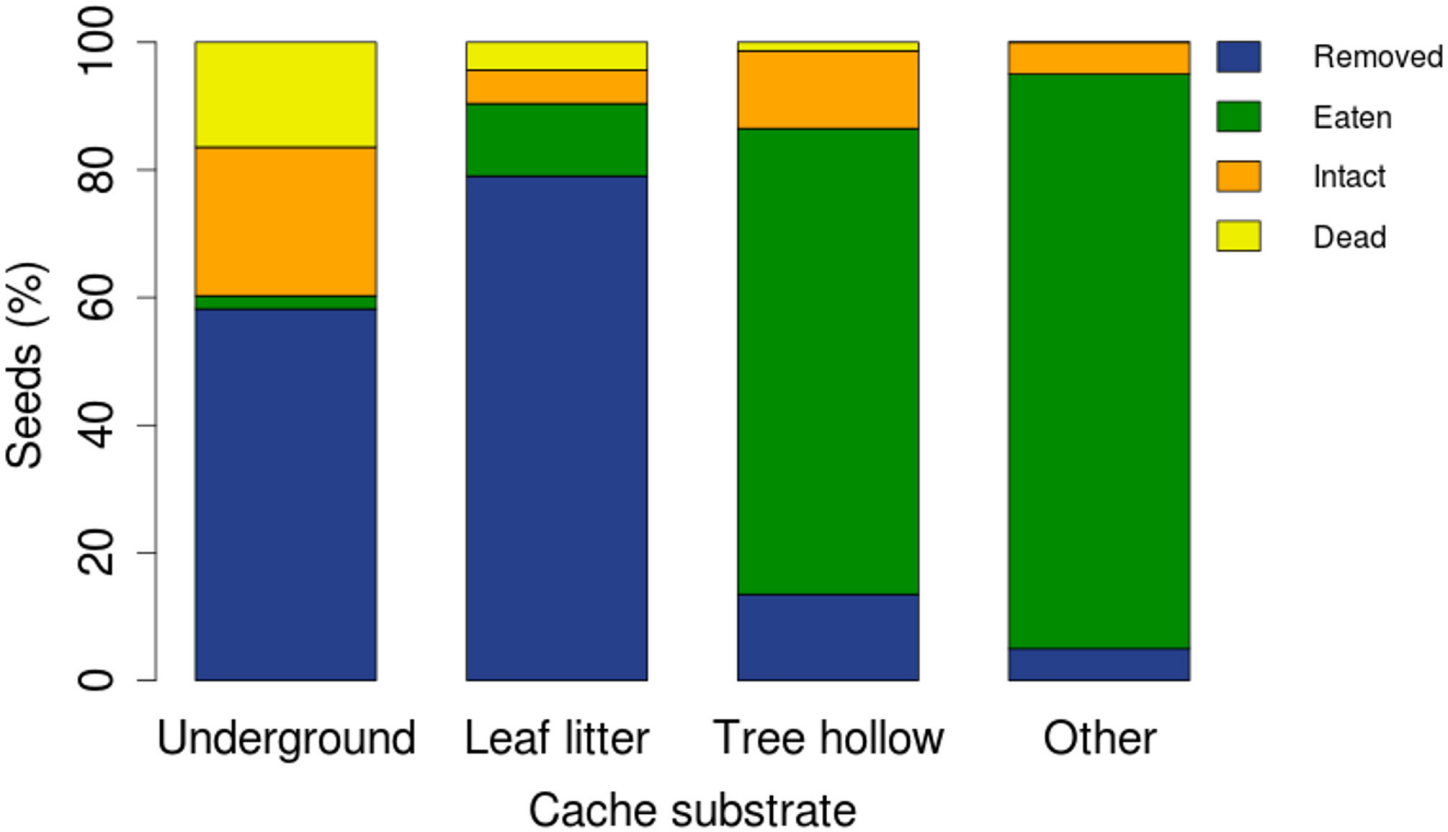
Cache strata and seed fate. Seed fates inside caches made in different substrates: underground, leaf-litter, tree hollow, and other substrates such as inside buttresses, and fallen logs and snags. Underground caches (n = 147) include seeds cached by the cricket, *Brachytrupes* species. Leaf-litter caches (n = 228) include seeds cached by murid rodents and hoary-bellied squirrel. Tree hollow caches (n = 74) and other substrate caches (n = 40) include seeds cached by murid rodents. 'Removed' seeds include seeds that were removed from the primary caches, 'eaten' seeds include seeds that were preyed upon, 'intact' seeds include seeds that were either viable or germinated, and 'dead' seeds include seeds that died due to invertebrate attack or fungal infestation.

Several seeds found in primary caches were removed (204 of 489 seeds), where the tags were cut off by the animal before moving the seeds which resulted in loss of seeds after the primary cache stage. A high percentage of seeds that were cached under leaf-litter by murid rodents and squirrels (87%) or buried underground by the cricket (43%) were removed again from the primary caches (Fig 6). However, we were able to locate only a single seed to its secondary cache before it was removed from this site again. The distance travelled from original location to primary cache by the seed was 4.4 m and the distance from primary to secondary cache was 8 m. An overall 57 seeds (11.7%) remained intact or germinated in primary caches; of these 34 seeds were in *Brachytrupes* caches (23%) (Table 3). The highest mortality of seeds was observed in murid rodent caches, which is possibly due to the high predation of seeds that were cached inside tree hollows (73%) and other substrates (93%) such as inside buttresses and fallen logs/snags (Table 3, Fig 6).

## Discussion

Our findings show that rodents can have significant impacts on post-dispersal seed fate and plant recruitment since they either removed or preyed upon more than half of the over 10,000 seeds that were monitored. In addition, more seeds were removed (cached) than immediately preyed upon, suggesting that secondary seed dispersal by rodents is an important mode of dispersal in this forest. The study supports earlier work, from other regions in the Paleotropics, on the role played by murid rodents in secondary seed dispersal [21, 24, 46]. Another novel and important finding of our study is the role of a cricket species from the genus *Brachytrupes* in seed caching, although seed caching by the cricket was restricted to the seeds of three tree species and mainly occurred in the rainy season. A high percentage of seeds cached by the cricket species germinated. The results are important given that 78% of the tree community in the study site is considered to be animal-dispersed with birds as the primary dispersal agent for 42% of the tree species [38]. Primary dispersal is known to be important for species that show lower recruitment near parent trees [47]. Three sympatric hornbill species are known as important primary dispersal agents for six of our study species (*Actinodaphne obovata*, *Litsea* sp., *Chisocheton cumingianus*, *Beilschmiedia assamicus*, *Prunus ceylanica*, *Horsfieldia kingii*) [38, 48], with the first three species showing reduced recruitment in anthropologically disturbed sites [40, 49]. Although hornbills are the main dispersal agents in these forests, especially for large-seeded species, they are also known to disperse seeds in clumps at nest trees and roost trees, which reduce seed survival and seedling recruitment [27, 28, 50]. While effective dispersal depends on whether more seeds get dispersed into suitable microsites for germination, the dispersal sites of these frugivores differ in the breeding and non-breeding season and impact the seed-fall patterns of consumed food plants [51, 52]. The previous study [28] had found that seeds of some species experienced high rates of seed predation by rodents at their nest sites. In this study, we found high rates of seed removal by murid rodents and some predation by porcupines, demonstrating that different rodent groups influence post-dispersal seed fates (and therefore, the recruitment) of several animal-dispersed plant species in different ways (either through removal or predation).

### Seed removal and predation: patterns within the tree community

We found that the rates of seed removal were over four times higher than rates of seed predation, suggesting a relatively larger role of seed removal in thinning out conspecific competition near parent trees [13, 53, 54, 55].

Our results for some of the tree species contrast with the findings of a previous study for the same species which found low rates of seed caching (< 2%) by rodents and high rates of on-site seed predation [29] at the same study site in 2007–2008. Velho *et al*. [28] found high rates of seed predation on *H*. *kingii* (96.2%, n = 280), *P*. *ceylanica* (78%, n = 230) and low rates of seed predation on *C*. *cumingianus* (1.9%, n = 250). In contrast, we found low rates of predation on *H*. *kingii* (8.1%, n = 1084)) and *P*. *ceylanica* (25.1%, n = 1087) and higher rates of seed predation on *C*. *cumingianus* (56.6%, n = 1002). The rates of seed removal also differed between the two studies. In the previous study, no seed removal was recorded for *C*. *cumingianus* and *H*. *kingii*, while 5.3% of *P*. *ceylanica* seeds were removed [28]. In contrast, we observed 39.3%, 61.2%, and 17.5% of seed removal for *C*. *cumingianus*, *H*. *kingii* and *P*. *ceylanica*, respectively. Higher rates of seed removal and lower rates of seed predation during our study period suggest inter-annual variation in rodent activity and caching behavior. This inter-annual variation in seed fates of three of the same tree species monitored during different years suggests complex consequences for tree community dynamics.

Among all the tree species sampled, *A*. *obovata* had the highest rates of removal and clearly is a species preferred by rodents for caching. The strong preference for *A*. *obovata* with very high rates of seed removal by rodents is likely to have implications for its distribution patterns [42]. Our camera trap monitoring also captured high rates of seed removal for *C*. *cumingianus*, *H*. *kingii* and *T*. *hodgsonii*, consistent with the overall pattern from tagged seeds. However, some tree species (*C*. *resiniferum*, *B*. *assamica*) underwent lower or negligible rates of seed removal when compared to others (*A*. *obovata*, *C*. *cumingianus*, *T*. *hodgsonii*), possibly reflecting differences in rodent food choice.

### Seed handling behavior

Rodent species showed clear differences in seed handling behaviors. Smaller-sized murid rodents, belonging to the genera *Niviventer* and *Rattus*, were the most frequent visitors on our seed plots. They always removed seeds from the seed plots and were never observed to eat seeds on-site. The two species of large-bodied porcupines were the main seed predators and never carried away seeds from seed plots, similar to findings from the earlier study [28]. An arboreal rodent, the hoary-bellied squirrel, was observed to prey upon as well as remove and cache seeds. However, the squirrel was observed to handle seeds of only two out of the ten tree species: small-seeded *T*. *hodgsonii* (always preyed upon on-site) and the large-seeded *C*. *cumingianus* (always removed). The hoary-bellied squirrel was earlier believed to be a pre-dispersal diurnal seed predator and was not observed to come to the ground often [41], but we recorded the species coming to the ground to consume and remove seeds.

The observation of a true cricket species from the genus *Brachytrupes* is the first known record of a cricket species caching seeds (> 15 mm in length) underground. There is evidence of seed dispersal by ants [2], and recently of a flightless grasshopper (weta, *Dienacrida rugosa*), which ingests fruits and passes several of the seeds unharmed, dispersing them in the process [56]. We observed that *Brachytrupes* sp. was active during the rainy season and cached seeds of only three species that fruited during these months: *Litsea* sp., *E*. *aristatus*, and *C*. *cumingianus*.

Emerald dove was observed to consume seeds of *T*. *hodgsonii*. Since the species is known to be predominantly a seed predator [57], it is likely that most of the consumed seeds were damaged.

### Factors affecting rates of seed removal and seed predation

In general, seed removal and predation rates are known to be higher on large-sized seeds as they provide greater resources to rodent seed predators [4, 58]. However, evidence from the Paleotropics is inconclusive; one study found support for this while another found low removal of large seeds [33, 59]. We tested the effect of seed traits (seed size and seed coat) on seed handling behavior and found support for our hypothesis that rodent choice and seed handling behavior will be influenced by seed traits. We found that large seeds underwent higher predation rates (by porcupines) but seed removal decreased with seed size which was due to reduced removal of the largest seeds (by murid rodents). We found support for our first prediction that seed selection (for predation or removal) will increase with seed size, but it was constrained by rodent body size. The seeds that were preferred by the rodents for removal were small-sized, similar to the findings of Wang and Chen [33], and this is likely due to the smaller body size of the murid rodents that hoard seeds in our study area. We also found that a hard seed-coat acted as a deterrent to on-site seed predation, but these seeds were preferred by murids for seed removal, especially when they were smaller. This confirms our second prediction that seed predation will decrease and seed removal increase with seed-coat thickness. A hard seed coat is likely to delay infestation by insects or fungus, thus improving the shelf life of a seed and making it more suitable for caching.

Seed removal is known to vary temporally with changing seed availability [11]. Although, inconclusive, we found that seed removal rates were higher in the intermediate seasons (September–October and March–April) but predation rates were generally low in all seasons, in a similar manner to a study in the Neotropics [30]. Conversely, another study [60] has reported higher rates of scatter-hoarding during resource-rich wet seasons, whereas Xiao *et al*. [59] have found consistent seed removal and predation across seasons in China and reported that innate seed traits were more important in influencing rates of seed removal and seed predation. This is likely to be true in our study site, where we found that small-seeded species, such as *A*. *obovata* and *T*. *hodgsonii*, were preferred by rodents for removal and also fruit in the intermediate seasons. Therefore, it is difficult to separate the effect of season from the effect of seed traits. However, seasonal effects may be important for seed removal because the small-sized murid rodents are known to have inter- and intra-annual fluctuations in their population size, and therefore, the effect of season needs to be further explored [61].

### Secondary seed dispersal and cache characteristics

Murid rodents were the dominant cache owners, followed by *Brachytrupes* sp. and hoary-bellied squirrel. Caches made by *Brachytrupes* sp. were always underground, those of hoary-bellied squirrel were always under a layer of leaf-litter, while murid rodent caches were present in a variety of substrate types. We observed that a small proportion of seeds were larder-hoarded inside cavities in tree trunks and buttresses by the murid rodents. Larder-hoarded seeds, which are often buried in deep substrates, are known to have lower rates of removal when compared to scatter-hoarded seeds, thus lowering the chance of survival of such seeds [5, 59]. We also observed that the seeds that were larder-hoarded by murid rodents had high rates of predation and low rates of removal.

However, over 88% of caches made by all species were single-seeded or double-seeded, which means that a majority of seeds being dispersed were scatter-hoarded. This is beneficial for tree species fitness as it reduces the chances of seeds being discovered by other foragers, and decreases the rates of fungal and insect infestations due to overcrowding of conspecific seeds [53, 62, 63]. *Brachytrupes* sp. transported seeds much closer to their original locations (usually < 1 m) when compared to murid rodents and hoary-bellied squirrel which transported seeds farther away (> 5 m) and carried most seeds up to 10 m from their original location, with maximum distance being 19 m. Over 80% of seeds were either removed, remained viable or germinated in underground caches made by the cricket or in leaf-litter caches made by the murid rodents and the squirrel. It appears that the deep burial of seeds in *Brachytrupes* caches provides optimum conditions for their germination. Overall, however, rodents removed far more seeds of many more species when compared to *Brachytrupes* sp. and were active during all seasons through the year. Removal of a large proportion of seeds from the primary cache, most likely suggests their movement into higher order caches as observed by Vander Wall *et al*. [1]. Once removed from primary or first order caches, we were unable to track all but a single seed to a secondary cache, during which the seed travelled approximately 12 m from the seed plot, supporting the existence of multiple-steps in a seed's movement path. Using radio-tagged seeds, Jansen *et al*. [13] showed that primary dispersal distances under-represent true dispersal distances as rodents re-cache seeds multiple times, depositing them much further away from primary dispersal sites each time.

Our study showed that rodents exhibited differential rates of seed caching and seed predation by following the seed fates of several tree species within the tree community. We found that seed fates were dependent on certain traits such as mass of the seed and the presence of a mechanical barrier. Our study also shows that the two main groups of terrestrial rodents (murid and hystricid) handle seeds very differently, with small-bodied murid rodents mainly caching seeds, while the two larger porcupine species preyed upon seeds on-site. We also report for the first time, evidence of seed caching by a species of true cricket. Our study adds new insights to the current knowledge of secondary seed dispersal in Asian forests.

## Supporting Information

S1 Table Experiment dates. Dates when the seed fate experiments were set-up near to (parent trees) and far from (random forest location) the parent trees for the study species. The experiments were carried out for 50 days, or until time of germination, or complete removal/predation of the seeds, whichever was first. We also carried out camera trap surveys simultaneously along with the seed fate experiments.
(DOC)

S2 Table Camera trapping effort. Number of seeds sampled, number of seed plots where camera traps were set, and number of camera trap nights distributed over seven study tree species during September 2011 –June 2012. The number of visits (and number of seeds handled) by all rodent species is provided. Camera trapping surveys were carried out for seven out of ten study tree species as the camera traps used for the other three species malfunctioned.
(DOC)

S1 Image A photograph of *Brachytrupes* sp., the true cricket that was observed caching seeds. (JPG)

S1 Video A murid rodent removing seeds from a seed plot. (MP4)

S2 Video A pair of hoary-bellied squirrels eating seeds inside a seed plot. (MP4)

S3 Video A pair of Himalayan crestless porcupines eating seeds inside a seed plot. (MP4)
