# Supplementary material for: Tracking Seed Fates of Tropical Tree Species: Evidence for Seed Caching in a Tropical Forest in North-East India
Source: PLoS One. 2015 Aug 6;10(8):e0134658. doi: 10.1371/journal.pone.0134658 (PMC4527596; doi:10.1371/journal.pone.0134658)
Supplement: S1 Table — Dates when the seed fate experiments were set-up near to (parent trees) and far from (random forest location) the parent trees for the study species. The experiments were carried out for 50 days, or until time of germination, or complete removal/predation of the seeds, whichever was first. We also carried out camera trap surveys simultaneously along with the seed fate experiments. (DOC) [file pone.0134658.s001.doc]

| Tree species | Seeds sampled | Seed plots | Camera trap nights | Murid rodent | Himalayan crestless porcupine | Brush-tailed porcupine | Hoary-bellied squirrel |
| --- | --- | --- | --- | --- | --- | --- | --- |
| *Actinodaphne obovata* | 146 | 11 | 44 | 126 (117) | 2 (0) | 0 (0) | 0 (0) |
| *Beilschmiedia assamica* | 299 | 15 | 186 | 28 (0) | 13 (1) | 0 (0) | 3 (0) |
| *Chisocheton cumingianus* | 374 | 15 | 188 | 17 (0) | 21 (224) | 1 (2) | 77 (74) |
| *Canarium resiniferum* | 527 | 10 | 156 | 26 (7) | 0 (0) | 9 (0) | 0 (0) |
| *Horsfieldia kingii* | 460 | 14 | 215 | 245 (143) | 2 (5) | 1 (0) | 1 (0) |
| *Prunus ceylanica* | 396 | 14 | 230 | 344 (81) | 18 (43) | 8 (1) | 7 (0) |
| *Talauma hodgsonii* | 533 | 9 | 63 | 75 (171) | 3 (0) | 0 (0) | 8 (79) |
