# Supplementary material for: Tracking Seed Fates of Tropical Tree Species: Evidence for Seed Caching in a Tropical Forest in North-East India
Source: PLoS One. 2015 Aug 6;10(8):e0134658. doi: 10.1371/journal.pone.0134658 (PMC4527596; doi:10.1371/journal.pone.0134658)
Supplement: S2 Table — Number of seeds sampled, number of seed plots where camera traps were set, and number of camera trap nights distributed over seven study tree species during September 2011 –June 2012. The number of visits (and number of seeds handled) by all rodent species is provided. Camera trapping surveys were carried out for seven out of ten study tree species as the camera traps used for the other three species malfunctioned. (DOC) [file pone.0134658.s002.doc]

| Tree species | Parent trees | Random forest location |
| --- | --- | --- |
| *Chisocheton cumingianus* | 12 May 2012 | 12 May 2012 |
| *Elaeocarpus aristatus* | 28 June 2011 | 28 June 2011 |
| *Turpinia pomifera* | 27 August 2011 | 27 August 2011 |
| *Litsea* sp. | 22 July 2011 | 22 July 2011 |
| *Actinodaphne obovata* | 28 September 2011 | 28 September 2011 |
| *Talauma hodgsonii* | 6 November 2011 | 9 November 2011 |
| *Horsfieldia kingii* | 18 March 2012 | 20 March 2012 |
| *Beilschmiedia assamica* | 4 November 2011 | 4 November 2011 |
| *Canarium resiniferum* | 13 December 2011 | 17 December 2011 |
| *Prunus ceylanica* | 16 January 2012 | 16 January 2012 |
