# Supplementary figures and images for: Tracking Seed Fates of Tropical Tree Species: Evidence for Seed Caching in a Tropical Forest in North-East India
Source: PLoS One. 2015 Aug 6;10(8):e0134658. doi: 10.1371/journal.pone.0134658 (PMC4527596; doi:10.1371/journal.pone.0134658)

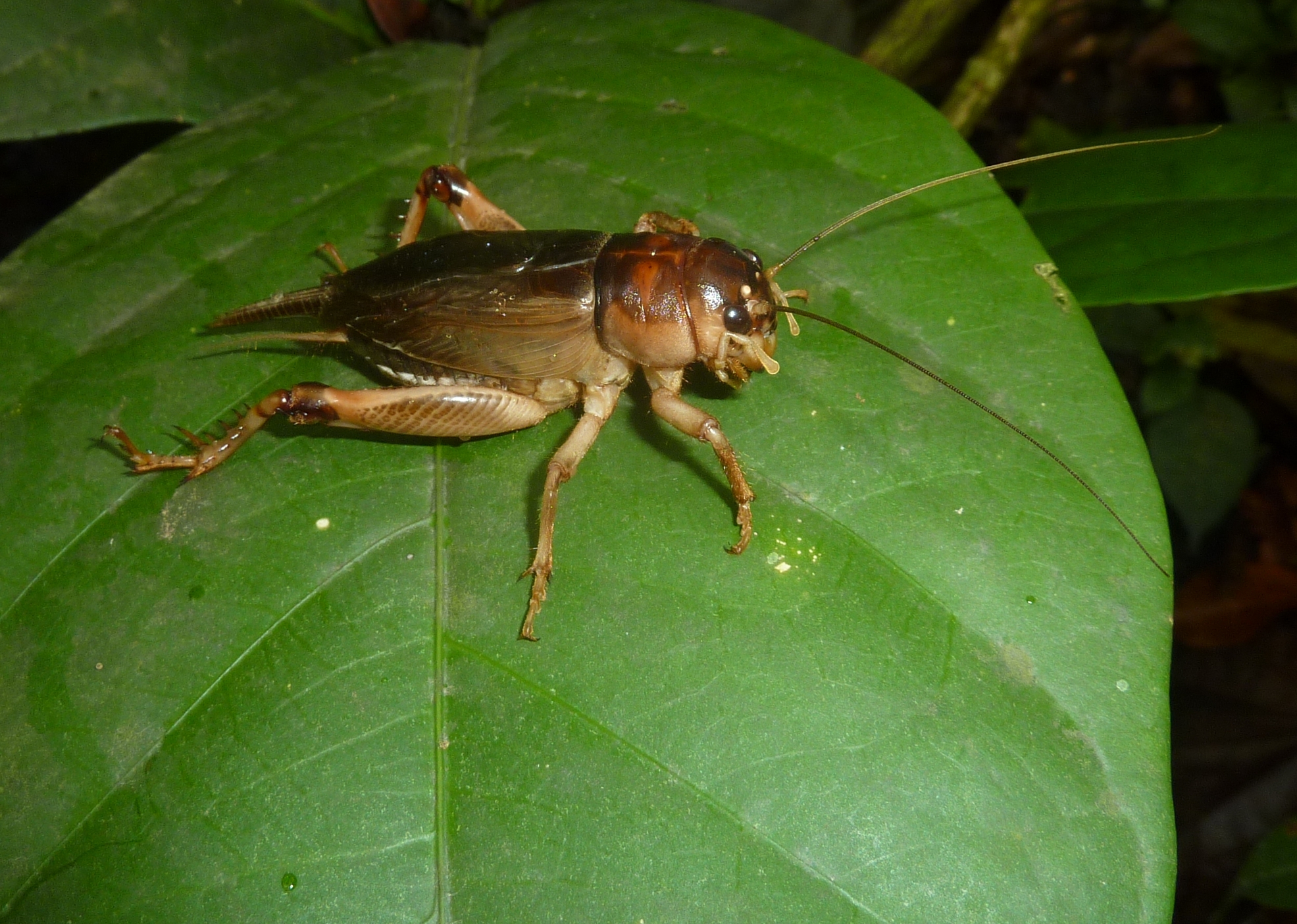

Supplement: S1 Image — (JPG) [file pone.0134658.s003.jpg]
